# Entropy optimized flow of Sutterby nanomaterial subject to porous medium: Buongiorno nanofluid model

**DOI:** 10.1016/j.heliyon.2023.e17784

**Published:** 2023-06-29

**Authors:** Shuguang Li, M. Ijaz Khan, Adel Bandar Alruqi, Sami Ullah Khan, Sherzod Shukhratovich Abdullaev, Bandar M. Fadhl, Basim M. Makhdoum

**Affiliations:** a School of Computer Science and Technology, Shandong Technology and Business University, Yantai, 264005, China; b Department of Mechanical Engineering, Lebanese American University, Kraytem, Beirut 1102-2801, Lebanon; c Department of Mathematics and Statistics, Riphah International University I-14, Islamabad 44000, Pakistan; d Department of Physics, Faculty of Science, King Abdulaziz University, Jeddah, Saudi Arabia; e Department of Mathematics, Namal University, Mianwali 42250, Pakistan; f Researcher, Faculty of Chemical Engineering, New Uzbekistan University, Tashkent, Uzbekistan; g Researcher of Scientific Department, Tashkent State Pedagogical University Named After Nizami, Tashkent, Uzbekistan; h Mechanical Engineering Department, College of Engineering and Islamic Architecture, Umm Al-Qura University, P. O. Box 5555, Makkah 21955, Saudi Arabia

**Keywords:** Sutterby nanofluid, Entropy generation, Thermophoresis, Thermal radiation, Brownian motion, Ohmic heating and chemical reaction

## Abstract

Owing to enhanced thermal impact of nanomaterials, different applications are suggested in engineering and industrial systems like heat transfer devices, energy generation, extrusion processes, engine cooling, thermal systems, heat exchanger, chemical processes, manufacturing systems, hybrid-powered plants etc. The current communication concerns the optimized flow of Sutterby nanofluid due to stretched surface in view of different thermal sources. The investigation is supported with the applications of external heat source, magnetic force and radiative phenomenon. The irreversibility investigation is deliberated with implementation of thermodynamics second law. The thermophoresis and random movement characteristics are also studied. Additionally, first order binary reaction is also examined. The nonlinear system of the governing problem is obtained which are numerically computed by s method. The physical aspects of prominent flow parameters are attributed graphically. Further, the analysis for entropy generation and Bejan number is focused. It is observed that the velocity profile increases due to Reynolds number and Deborah number. Larger Schmidt number reduces the concentration distribution. Further, the entropy generation is improved against Reynolds number and Brinkman parameter.

## 1 Introduction

The heat transportation has tremendous applications in various fields like fuel cells, computers processor, air-conditioners, hybrid engines, and many others. In effective transportation of heat low heat conductivity of working fluids is a barrier. Heat transport phenomena of conventional liquids can be improved by mixing nano-size (1−100nm) solid particles in base liquids [1,2]. The model to study heat transfer enhancement in nanofluids is provided by Buongiorno [3]. He reported that thermophoresis and random diffusion are ruling factors among other factors. Radiative nanomaterial flow with Lorentz force impact is exemplified by Prasad et al. [4]. Heat analysis in convectively heated magnetohydromagnetic nanoliquid flow is illustrated by Tian et al. [5]. Thermal transfer effect in convective hybrid nanoliquid flow with radiation effect is illuminated by Hayat et al. [6]. Heat analysis in Maxwell nanomaterials flow subject to rotating porous medium is considered by Ahmad et al. [7]. Entropy aspects in Carreau nanomaterial flow is reported by Khan et al. [8]. Chu et al. [9] reported the thermal determination of nanofluid with viscoelastic material. Adnan et al. [10] reported the Riga surface flow for assessing the freezing thermal assessment of nanofluid. Ibrahim et al. [11] determined the correlations for neural network associated to the nanofluid problem. Akbar and Khan [12] presented the ciliated nanofluid flow under the viscous heating feature. Khan et al. [13] reported the triple diffusion impact of nanofluid in horizontal plate flow. The nanoparticles interaction in Y-shaped obstacle with FEM simulations are deducted by Khan et al. [14]. Sharma et al. [15] focused on the assessment of entropy generation for couette nanofluid flow. The heat source applications with entropy generation impact in channel was observed by Sharma et al. [16].

The heat and mass transport processes subject to chemical reaction have received notable consideration now a days due to tremendous applications in different process including cleaning of materials, soap reaction, pulp and drying etc. Chemically reactive flow over a porous vertical sheet is analyzed by Postelnicu [17]. Thermal characteristic in chemically reactive nanomaterial flow subject to heated sheet is reported by Rout et al. [18]. Radiation impact in reactive magnetohydrodynamic nanoliquid flow due to porous plate is studied by Reddy et al. [19]. Heat transfer effect in reactive flow of third grade liquid with Lorentz force is explored by Hayat et al. [20]. Due to diverse characteristics of nanofluid they cannot be completely expressed by a single constitutive relation. These materials include clays, hydro-genased caster oil, paints, butter, shampoos, and jam etc. Furthermore, various models for non-Newtonian fluid are mentioned in Refs. [21,22,23,24,25,26,27,28,29,30]. Bejan [31,32] studied the entropy rate in convectively heated flow. Irreversibility in radiative Sisko liquid flow with dissipation towards stretched surface is highlighted by Khan et al. [33]. Entropy analysis in steam flow with turbine blades and volumetric heating is surveyed by Vatanmakan et al. [34]. Activation energy effect in magnetohydrodynamic Casson nanoliquid flow subject to entropy is analyzed by Hayat et al. [35]. Irreversibility analysis in viscoelastic Poiseuille nanofluid flow is described by Gul et al. [36]. Randomness analysis for MHD nanomaterial flow is discussed by Xie and Jian [37]. Irreversibility in viscous nanoliquid flow with forced convection is scrutinized by Khan et al. [38]. Irreversibility analysis in nanoliquid flow is reported by Huminic and Huminic [39]. Xiang et al. [40,41,42] worked on micro-channel fluid flow in the presence of thermal performance of diode resistance and heat sink. Dai et al. [43], Zhu et al. [44] and Bian et al. [45] respectively worked on coupled peri-dynamics smoothed particle fluid structure analysis, generalized micro-fluidic rectifiers and bio-inspired magnetism responsive hybrid microstructures towards liquid droplet. Recently, Du et al. [46], Fan et al. [47] and Qu et al. [48] explored the applications of fluids in the presence of different nanomaterials. In couple of decades, frequent researchers concentrate on heat transfer problem, surface pressure in the presence of metalic and non-metalic materials; i. e., hydrate-bearing sediment within a granular thermodynamic system [49], temperature and pressure dependent pore micro-structures [50], stress and deformation analysis with temperature field [51], colloids transport in 2D permeable media [52], pressure pulsation attributes of centrifugal pump [53] and energy relaxation of hot electrons via coupling with optical [54]. Some other important and fruitful work on the modeling of fluid flow is listed in Refs. [55,56,57,58,59,60].

After highlighting the detailed research on nanomaterials, it is observed that different researchers have reported the heat transfer phenomenon due to interaction of nanofluids with various flow configurations. However, the thermal applications of Sutterby nanomaterial with entropy generation impact are not focused yet. The major aspect of current model is summarized as:

➢ The heat and mass transfer phenomenon due to chemically reactive Sutterby nanomaterial has been investigated.
➢ The flow problem is observed with assessment of external heat source, viscous dissipation and Joule hating impact.
➢ The irreversibility analysis is predicted for Sutterby nanofluid problem.
➢ The linear thermal radiation and chemical reaction features are focused.
➢ The investigation for Bejan number is reported to optimized phenomenon.
➢ The impact of physical parameters on concentration phenomenon, fluid flow, Bejan number, thermal field and entropy rate are examined. Moreover, numerical computations are performed for drag force, solutal transport rate and gradient of temperature.

It is remarked that source of flow is stretching surface which is an interesting flow configuration. The heat transfer phenomenon due to stretching surface play important applications in hot rolling, plastic films, extrusion processes, crystal growing, paper production etc.

## 2 Statement of problem

A two-dimension steady flow of Sutterby nanomaterial flow by a stretched permeable surface is considered. Heat source, viscous dissipation, magnetic force and radiation are accounted in heat equation. Brownian and thermophoresis motion are addressed. Entropy rate is calculated. Chemical reaction at catalytic surface is focused. Magnetic force of intensity ($B_{0}$) is employed. Suppose that $u_{w}=ax$ is stretching flow along with a>0.

Eq. (1) defining the Cauchy stress tensor [30]:

(1) τ=−pI+S

For Sutterby fluid model, defining extra stress tensor S is defined in Eqs. (1), (2), (3), (4), (5) as follows [30,59]:

(2) $$S=\mu_{0}{\left(\frac{\sinh^{-1}\;B\sqrt{{\left(\left|\gamma^{•}\right|\right)}^{2}}}{B\sqrt{{\left(\left|\gamma^{•}\right|\right)}^{2}}}\right)}^{m}\left|\gamma^{•}\right|$$

(3) $$\left|\gamma^{•}\right|=\sqrt{\frac{1}{2}trace{\left(A_{1}\right)}^{2}}$$

(4) $$A_{1}=L+L^{T}$$

(5) L=∇V

For two dimensional flow, the flow equations for Sutterby fluid model are [30,59]:

(6) $$\frac{\partial u}{\partial x}+\frac{\partial v}{\partial y}=0\text{,}$$

(7) $$u\frac{\partial u}{\partial x}+v\frac{\partial u}{\partial y}=\frac{\mu }{\rho }\frac{\partial }{\partial y}\left[\frac{\partial u}{\partial y}+\frac{mB^{2}}{2}{\left(\frac{\partial u}{\partial y}\right)}^{3}\right]-\frac{\sigma B_{0}^{2}}{\rho }u-\frac{\mu }{\rho k^{*}}u\text{,}$$

(8) $$\begin{array}{l} u\frac{\partial T}{\partial r}+w\frac{\partial T}{\partial z}=\frac{k_{f}}{\rho C_{p}}\left(1+\frac{16\sigma^{*}T_{\infty }^{3}}{3k_{f}k_{1}}\right)\frac{\partial^{2}T}{\partial y^{2}}+\tau \left[D_{B}\frac{\partial C}{\partial y}\frac{\partial T}{\partial y}+\frac{D_{T}}{T_{\infty }}{\left(\frac{\partial T}{\partial y}\right)}^{2}\right]+\\ \frac{\sigma B_{0}^{2}}{\rho C_{p}}u^{2}+\frac{\mu_{f}}{\rho C_{p}}{\left(\frac{\partial u}{\partial y}\right)}^{2}\left[1+\frac{mB^{2}}{6}{\left(\frac{\partial u}{\partial y}\right)}^{2}\right]+\frac{Q_{0}}{\rho C_{p}}\left(T-T_{\infty }\right)\text{,} \end{array}\}$$

(9) $$u\frac{\partial C}{\partial x}+v\frac{\partial C}{\partial r}=D_{B}\frac{\partial^{2}C}{\partial y^{2}}+\frac{D_{T}}{T_{\infty }}{\left(\frac{\partial T}{\partial y}\right)}^{2}-K_{r}^{2}\left(C-C_{\infty }\right)\text{,}$$

Eqs. (6), (7), (8), (9) are the governing equations of the problem satisfying following constraints:

(10) $$\begin{array}{l} u=u_{w}\left(x\right)=ax,v=0,T=T_{w},C=C_{w}\;\text{at}\;y=0\\ u\to 0,C\to C_{\infty },T\to T_{\infty },\text{at}\;y\to \infty \text{,} \end{array}\}\text{,}$$

New variables are defined as [51]:

(11) $$\begin{array}{l} u=axf^{\prime }\left(\eta \right),v=-\sqrt{a\nu }f\left(\eta \right),\eta =y\sqrt{\frac{a}{\nu },}\\ \theta \left(\eta \right)=\frac{T-T_{\infty }}{T_{w}-T_{\infty }},\varphi \left(\eta \right)=\frac{C-C_{\infty }}{C_{w}-C_{\infty }}\text{,} \end{array}\}\text{.}$$

Using Eq. (11), the following system of Eqs. (12), (13), (14), (15) is attained:

(12) $$\left(1+\frac{m}{2}DeRef^{\prime \prime^{2}}\right)\;f^{\prime \prime \prime }-Mf^{\prime }-\lambda f^{\prime }-f^{\prime^{2}}+ff^{\prime \prime }=0\text{,}$$

(13) $$\left(1+\frac{4}{3}R\right)\;\theta^{\prime \prime }+\mathrm{Pr}\;Nb\theta^{\prime }\varphi^{\prime }+\mathrm{Pr}\;Nt\theta^{\prime^{2}}+\mathrm{Pr}\;MEcf^{\prime^{2}}+\frac{1}{2}\mathrm{Pr}\;Ec\left[f^{\prime \prime^{2}}+\frac{m}{6}DeRef^{\prime \prime^{2}}\right]+\mathrm{Pr}\;\beta \theta +\mathrm{Pr}\;f\theta^{\prime }=0\}\text{,}$$

(14) $$\varphi^{\prime \prime }+\frac{Nt}{Nb}\theta^{\prime \prime }-Sc\gamma \varphi +Scf\varphi^{\prime }=0\text{,}$$

with

(15) $$\begin{array}{l} f\left(0\right)=0,\theta \left(0\right)=1,f^{\prime }\left(0\right)=1,\varphi \left(0\right)=1\text{,}\\ f^{\prime }\left(\infty \right)=0,\varphi \left(\infty \right)=0,\theta \left(\infty \right)=0\text{.} \end{array}\}$$

Here non-dimensional variables are $De\left(=B^{2}a^{2}\right)$, $Re\left(=\frac{ax^{2}}{\nu_{f}}\right)$, $M\left(=\frac{\sigma_{f}B_{0}^{2}}{\rho_{f}a}\right)$, $\mathrm{Pr}\left(=\frac{\mu_{f}c_{p}}{k_{f}}\right)$, $R\left(=\frac{4\sigma^{*}T_{\infty }^{3}}{k_{f}k_{1}}\right)$, $\lambda \left(=\frac{\nu_{f}}{ak^{*}}\right)$, $Nb\left(=\frac{\tau D_{B}\left(C_{w}-C_{\infty }\right)}{\nu_{f}}\right)$, $Nt=\left(\frac{\tau D_{T}\left(T_{w}-T_{\infty }\right)}{T_{\infty }\nu_{f}}\right)$, $Ec\left(=\frac{a^{2}x^{2}}{C_{p}\left(T_{w}-T_{\infty }\right)}\right)$, $\gamma \left(=\frac{k_{r}^{2}}{a}\right)$, $\beta \left(=\frac{Q_{0}}{{\left(\rho C_{p}\right)}_{f}a}\right)$ and $Sc\left(=\frac{\nu_{f}}{D_{B}}\right)$.

## 3 Quantities of interest

Drag force, solutal transport rate and Nusselt number are defined in Eq. (16) [59]:

(16) $$\begin{array}{l} Cf_{x}=\frac{-\tau_{w}}{\rho u_{w}^{2}},Nu_{x}=\frac{xq_{w}}{k\left(T_{w}-T_{\infty }\right)}\text{,}\\ Sh_{x}=\frac{xj_{w}}{k\left(C_{w}-C_{\infty }\right)}\text{,} \end{array}\}$$

Here $\tau_{w}$, shear stress, $j_{w}$ mass flux and $q_{w}$ heat flux are given in Eq. (17) [59]:

(17) $$\begin{array}{l} \tau_{w}=\mu {\left(\frac{\partial u}{\partial y}\right)}_{y=0}-\mu \frac{mB^{2}}{6}{\left(\frac{\partial u}{\partial y}\right)}_{y=0}^{3}\text{,}\\ j_{w}=-D_{B}{\left(\frac{\partial C}{\partial y}\right)}_{y=0},q_{w}=-k{\left(\frac{\partial T}{\partial y}\right)}_{y=0} \end{array}\}$$

(18) $$\begin{array}{l} Cf_{x}\sqrt{{Re}_{x}}=-\left[f^{\prime \prime }\left(0\right)+\frac{m}{6}DeRef^{\prime \prime^{3}}\left(0\right)\text{,}\right]\\ Nu_{x}{\left({Re}_{x}\right)}^{-\frac{1}{2}}=-\theta^{\prime }\left(0\right),Sh_{x}{\left({Re}_{x}\right)}^{-\frac{1}{2}}=-\varphi^{\prime }\left(o\right)\text{,} \end{array}\}$$

Eq. (18) is the dimensionless form expression (17).

## 4 Entropy generation calculation

The entropy generation is defined in Eqs. (19), (20) with support of certain laws

(19) $$E_{G}=\frac{k}{T_{\infty }^{2}}\left(1+\frac{4}{3}R\right)\;{\left(\frac{\partial T}{\partial y}\right)}^{2}+\frac{\mu }{T_{\infty }}{\left(\frac{\partial u}{\partial y}\right)}^{2}\left[1+\frac{mB^{2}}{6}{\left(\frac{\partial u}{\partial y}\right)}^{2}\right]+\frac{\mu }{T_{\infty }k^{*}}u^{2}+\frac{\sigma B_{0}^{2}}{T_{\infty }}u^{2}+\frac{R_{1}D_{B}}{C_{\infty }}{\left(\frac{\partial C}{\partial y}\right)}^{2}+\frac{R_{1}D_{B}}{T_{\infty }}\left(\frac{\partial T}{\partial y}\right)\;\left(\frac{\partial C}{\partial y}\right)\text{,}\}$$

We have

(20) $$S_{G}=\left(1+\frac{4}{3}R\right)\;\alpha_{1}\theta^{{}^{\prime }2}+B_{r}f^{\prime \prime^{2}}\left[1+\frac{m}{6}DeRef^{\prime \prime^{2}}\right]+B_{r}\lambda f^{\prime^{2}}+MB_{r}f^{\prime^{2}}+L\frac{\alpha_{2}}{\alpha_{1}}{\varphi^{\prime }}^{2}+L\theta^{\prime }\varphi^{\prime }\}\text{,}$$

The Bejan number is defined in Eq. (21) as [59]:

(21) $$Be=\frac{\left(1+\frac{4}{3}R\right)\;\alpha_{1}\theta^{{}^{\prime }2}+L\frac{\alpha_{2}}{\alpha_{1}}{\varphi^{\prime }}^{2}+L\theta^{\prime }\varphi^{\prime }}{\left(1+\frac{4}{3}R\right)\;\alpha_{1}\theta^{{}^{\prime }2}+B_{r}f^{\prime \prime^{2}}\left[1+\frac{m}{6}DeRef^{\prime \prime^{2}}\right]+B_{r}\lambda f^{\prime^{2}}+MB_{r}f^{\prime^{2}}+L\frac{\alpha_{2}}{\alpha_{1}}{\varphi^{\prime }}^{2}+L\theta^{\prime }\varphi^{\prime }}\text{.}$$

Here dimensionless parameters are $S_{G}=\frac{E_{G}T_{\infty }\nu_{f}}{ak_{f}\left(T_{w}-T_{\infty }\right)}$, $B_{r}=\frac{\mu_{f}a^{2}x^{2}}{k_{f}\left(T_{w}-T_{\infty }\right)}$, $L=\frac{R_{1}D\left(C_{w}-C_{\infty }\right)}{k_{f}}\text{,}$ $\alpha_{1}=\frac{T_{w}-T_{\infty }}{T_{\infty }}$ and $\alpha_{2}=\frac{C_{w}-C_{\infty }}{C_{\infty }}$.

## 5 Numerical simulations

The flow model presented in the dimensionless form is numerically computed with shooting technique. This method is based on conversion of higher order system into first order system. The motivation for using this scheme is due to excellent accuracy. Shooting method contains simple step instead of any complicated discretization like other schemes.

## 6 Validation of results

The validation of results is observed is Table 1 for limiting case. The numerical data is compared with the analytical work performed by Hayat et al. [60]. A fine assessment of obtained results is noticed.

**Table 1:** Comparative analysis for $f^{\prime \prime }\left(0\right)$ when m=M=0..

λ	Hayat et al. [60]	Present results
0.0	−1.000000	−1.000000
0.5	−1.224747	−1.224748
1.5	−1.581147	−1.581149
2.0	−1.732057	−1.7320577

## 7 Discussion

Different prominent parameters versus $\left(f^{\prime }\left(\eta \right)\right)$, (φ(η)), (Be), (θ(η)) and $\left(S_{G}\right)$ are scrutinized. Gradient of temperature, solutal transport rate and drag force for prominent variables are discussed.

### 7.1 Velocity profile

**Fig. 1:**
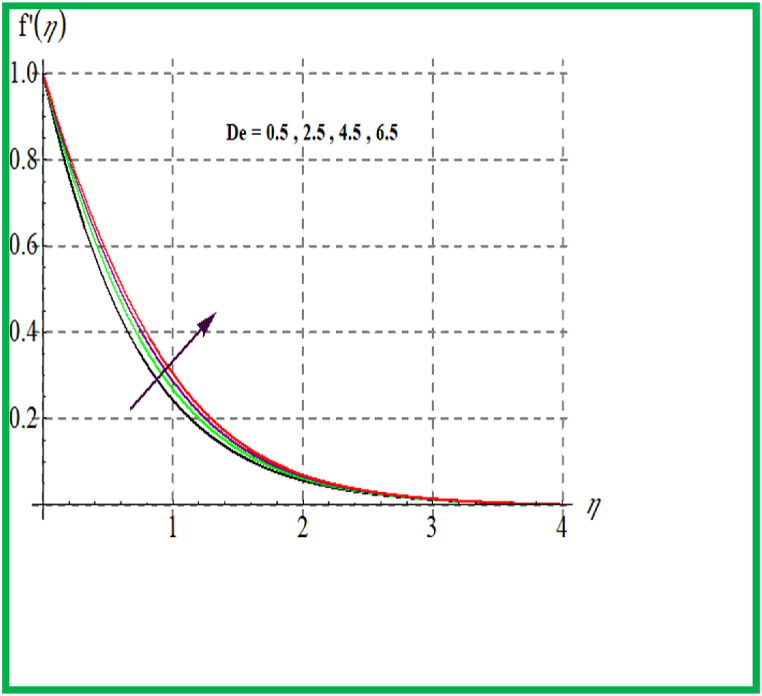
De versus $f^{\prime }\left(\eta \right)$.

**Fig. 2:**
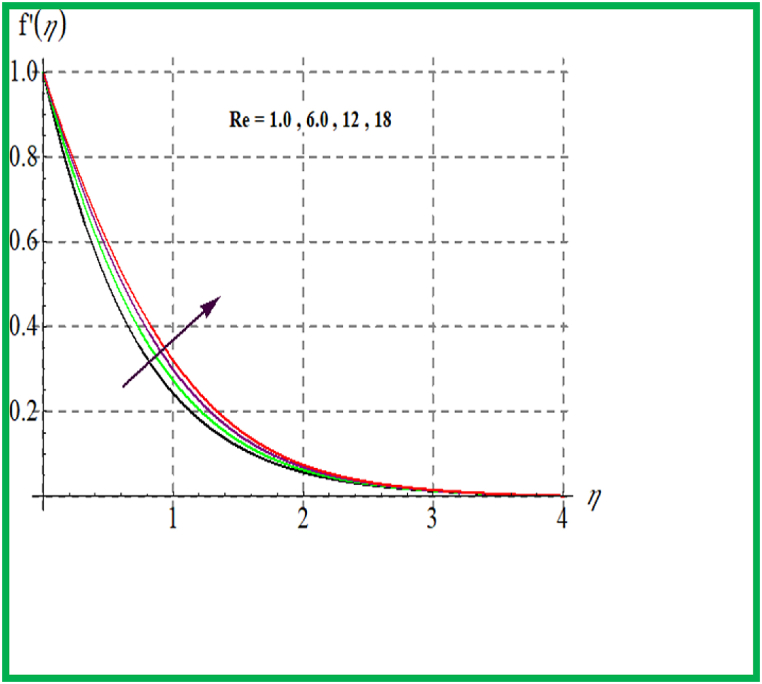
$f^{\prime }\left(\eta \right)$ for Re.

**Fig. 3:**
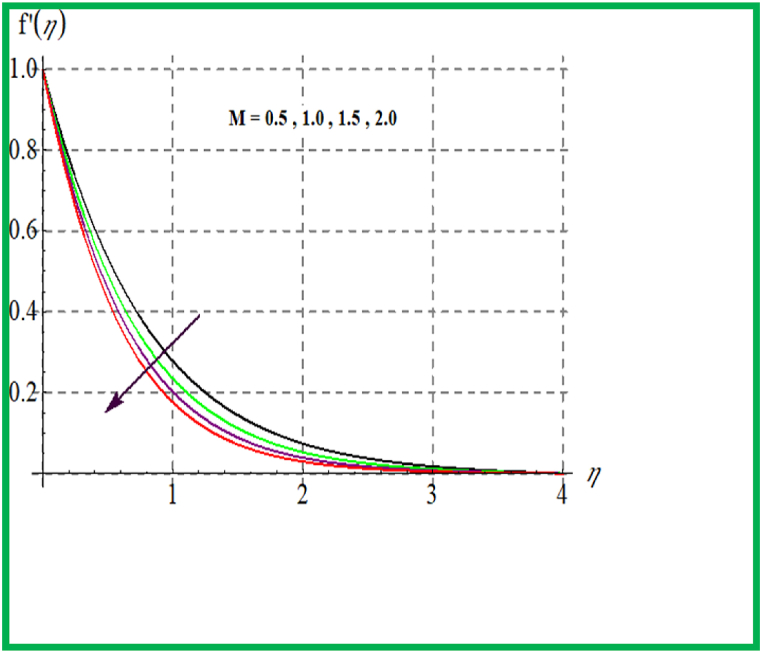
$f^{\prime }\left(\eta \right)$ for M.

**Fig. 4:**
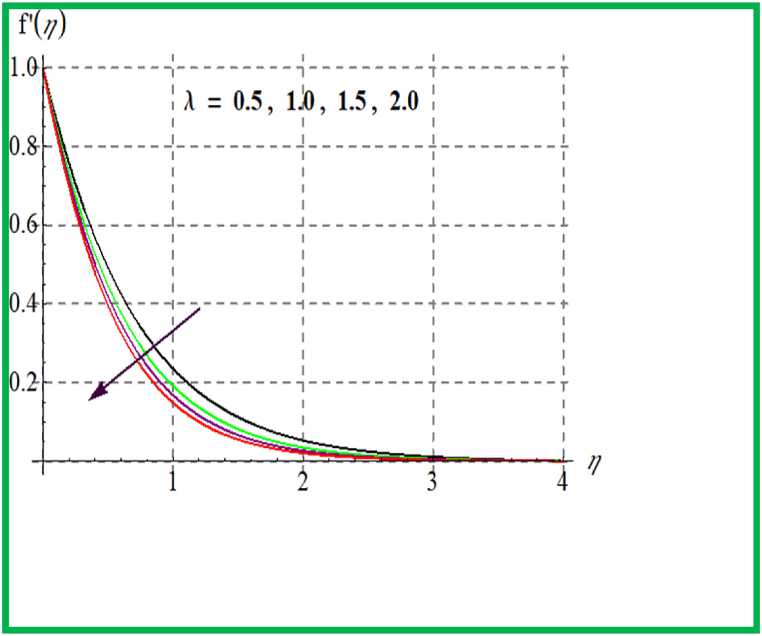
$f^{\prime }\left(\eta \right)$ for λ.

Influence of De on $f^{\prime }\left(\eta \right)$ is discovered in Fig. 1. Velocity upsurges for higher Deborah number De. In Fig. 2 Reynold number effect on $f^{\prime }\left(\eta \right)$ is checked. It is noticed that larger Reynold number (Re) improve velocity field. Physically larger Re reduces viscous forces and thus velocity increases. Fig. 3 depicts result of (M) on $f^{\prime }\left(\eta \right)$. Physically an increase in M augments resistive force in flow region and so velocity boosts up. In Fig. 4 porosity parameter versus $f^{\prime }\left(\eta \right)$ is plotted. Larger porosity variable reduces the velocity profile.

### 7.2 Temperature profile

**Fig. 5:**
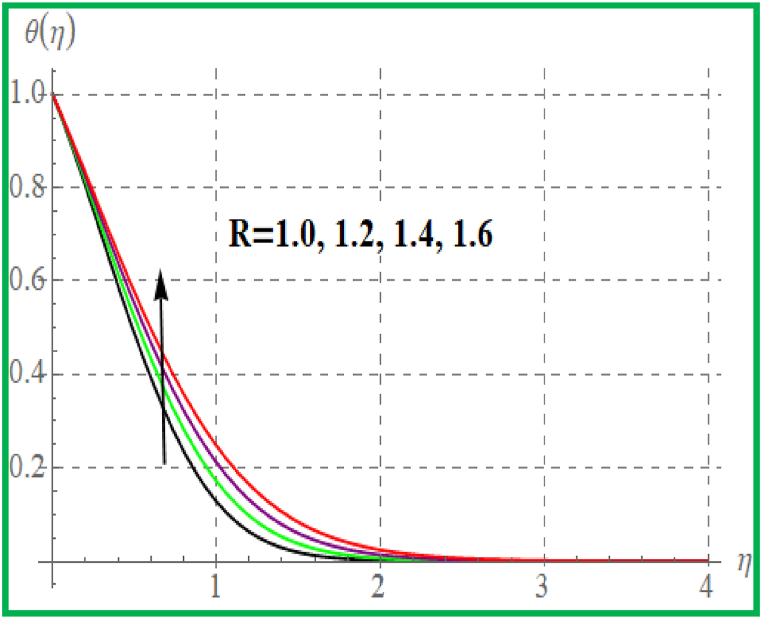
θ(η) for R.

**Fig. 6:**
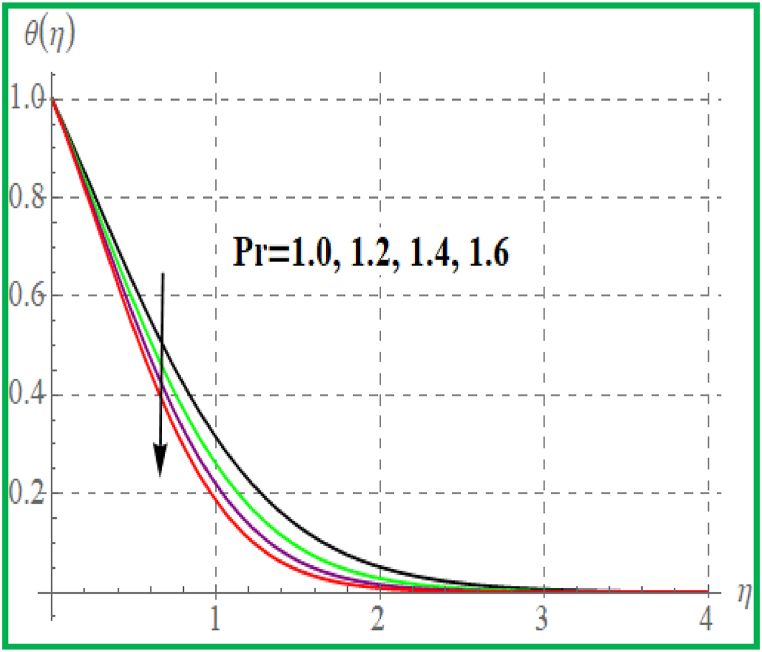
θ(η) for Pr.

**Fig. 7:**
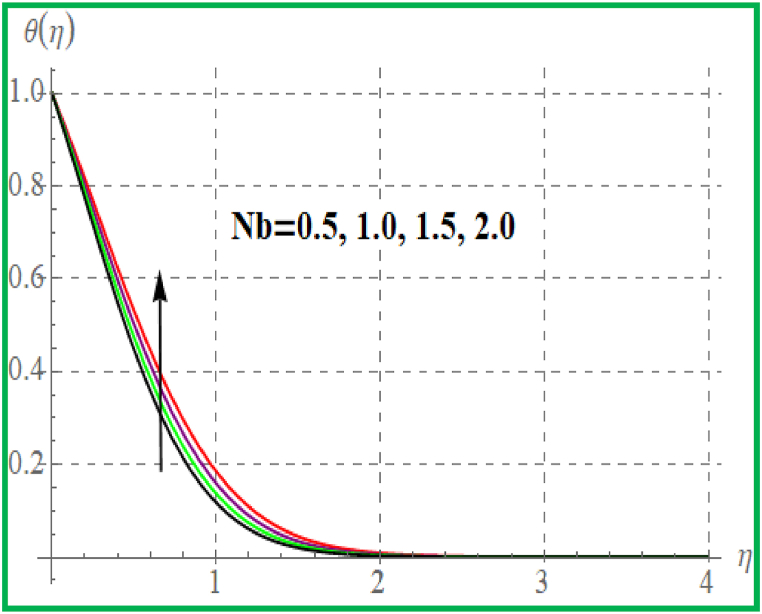
θ(η) for Nb.

**Fig. 8:**
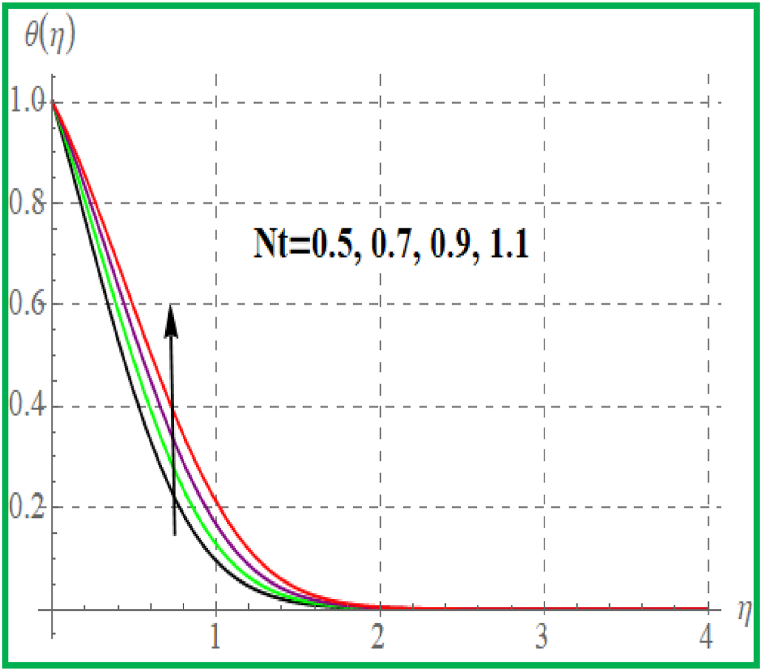
θ(η) for Nt.

**Fig. 9:**
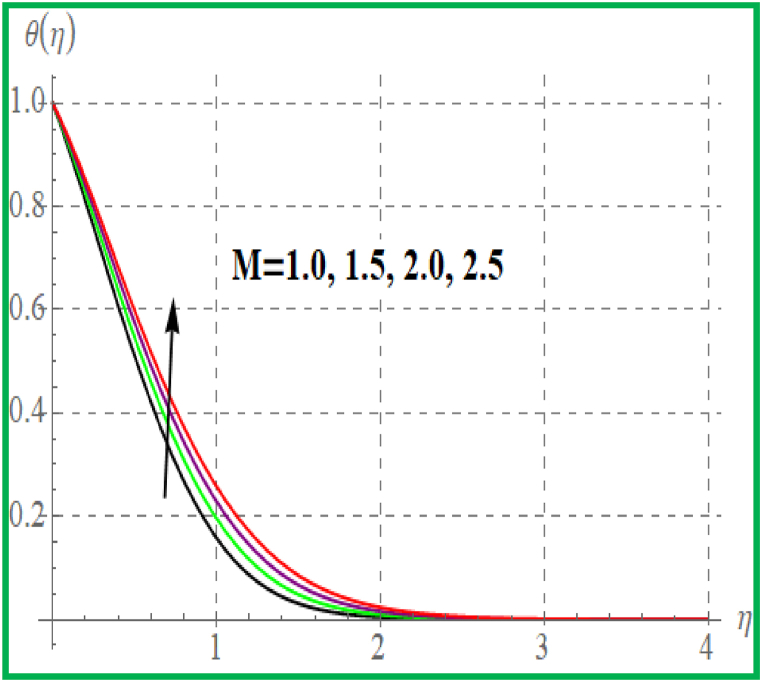
θ(η) for M.

Fig. 5 is sketched effect of radiation variable on temperature (θ(η)). Clearly θ(η) increases for higher radiation variable. Prandtl number effect on θ(η) is portrayed in Fig. 6. An enhancement in Pr temperature (θ(η)) reduces. Influence of temperature for Nb is portrayed in Fig. 7. For larger evaluation of Nb the (θ(η))improves. The Brownian motion is the random fluid particles movement for which the particles collide with each and as a result more heat transfer is predicted. Fig. 8 is outlined to check the effect of Nt on θ(η). Higher Nt improves the fluid temperature. Such effects are physically attributed to the thermophoresis phenomenon. Fig. 9 is plotted for temperature with variation of magnetic variable (M). Physically magnetic field produces more resistive force due to which more heat produces and thus θ(η) increases.

### 7.3 Concentration phenomenon

**Fig. 10:**
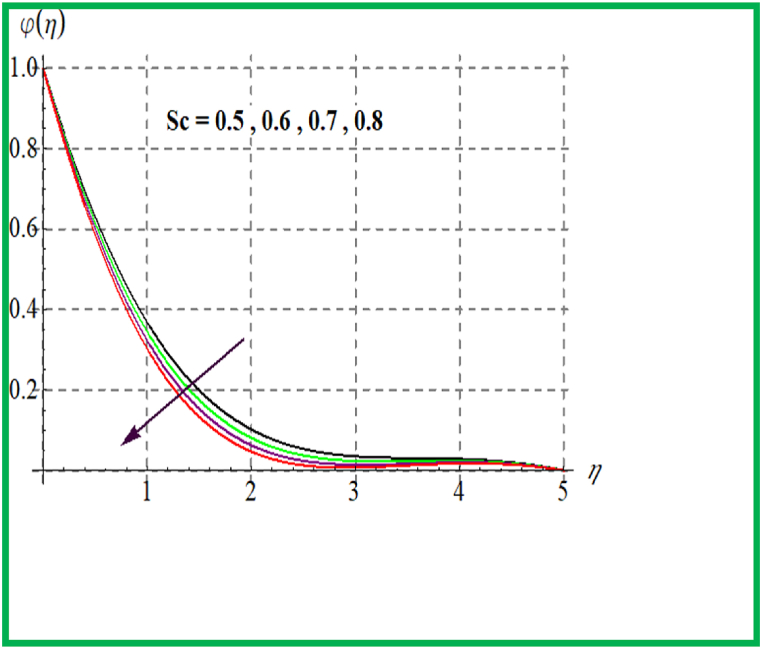
φ(η) for Sc.

**Fig. 11:**
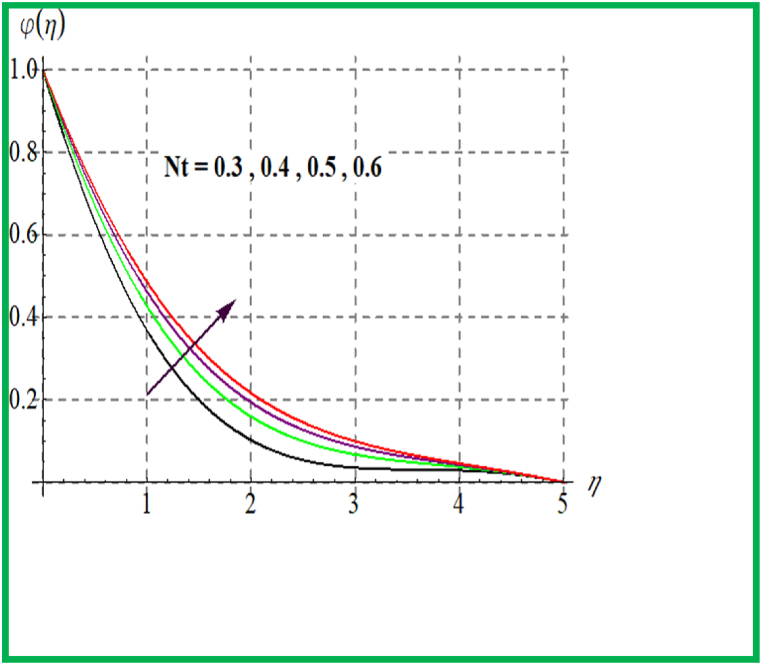
φ(η) for Nt.

**Fig. 12:**
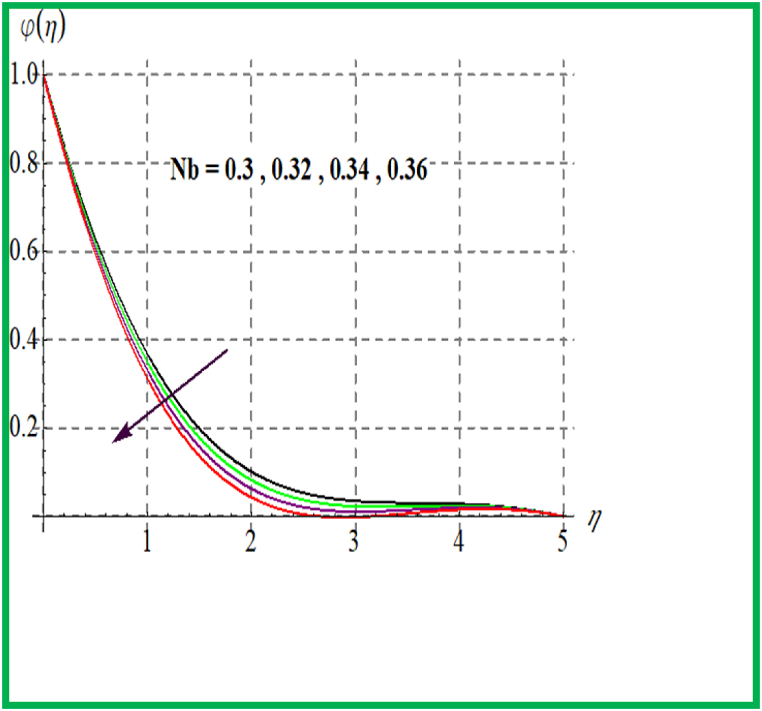
φ(η) for Nb.

**Fig. 13:**
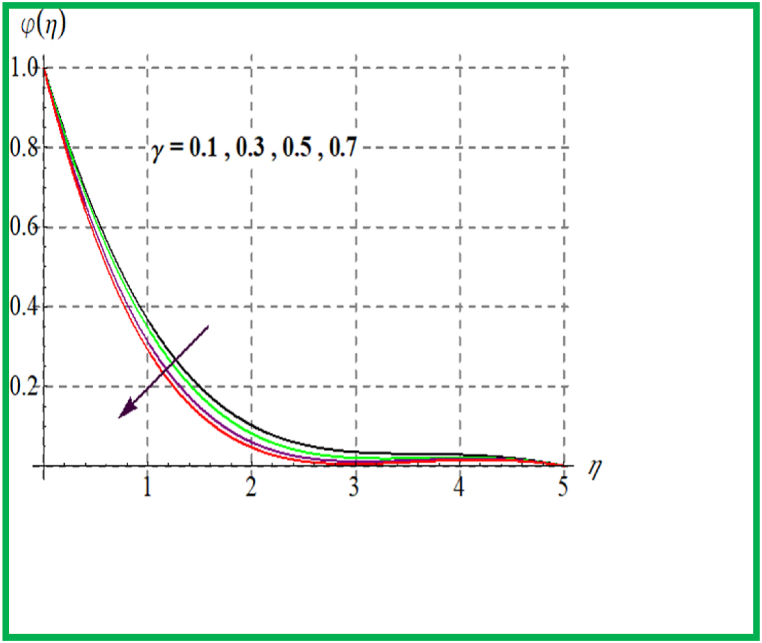
φ(η) for γ.

Performance of concentration for Sc is seen in Fig. 10. Physically solutal diffusivity decays for larger Schmidt number. Thus φ(η) is diminished. An opposite behavior is noted for φ(η) with variation in Brownian and thermophoresis variable (see Fig. 11, Fig. 12). Variation of φ(η) for (γ) is portrayed in Fig. 13. Higher reaction parameter leads to reduces concentration.

### 7.4 Bejan number and entropy rate

**Fig. 14:**
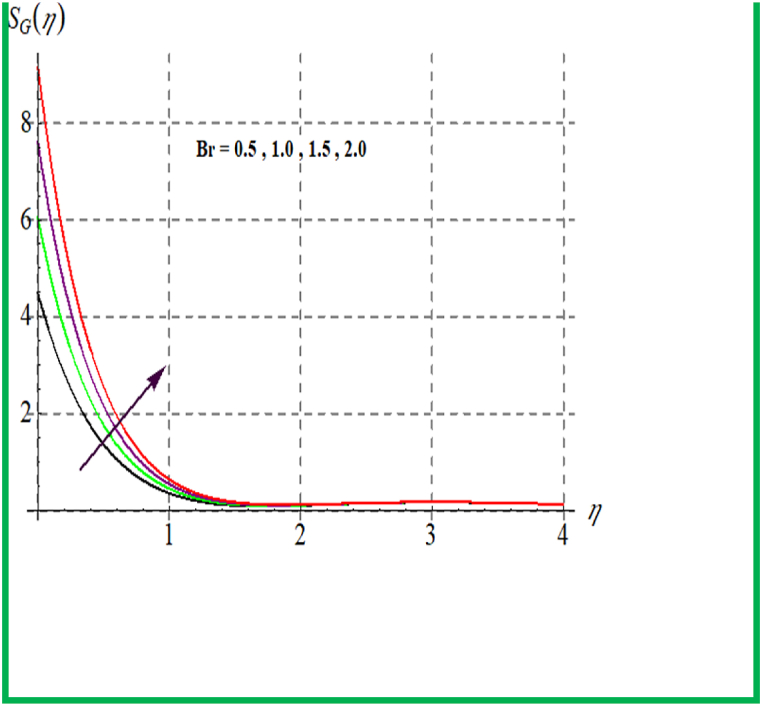
Br versus $N_{G}$.

**Fig. 15:**
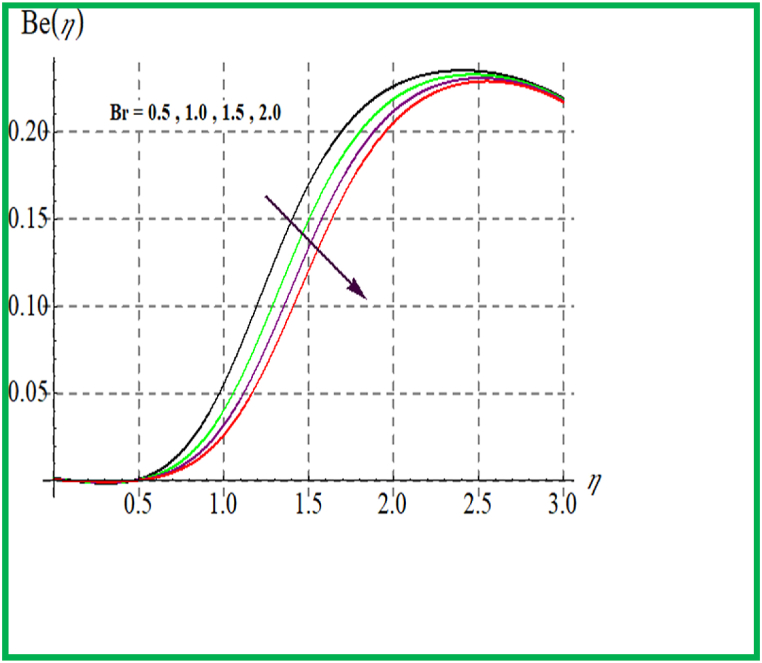
Br versus Be.

**Fig. 16:**
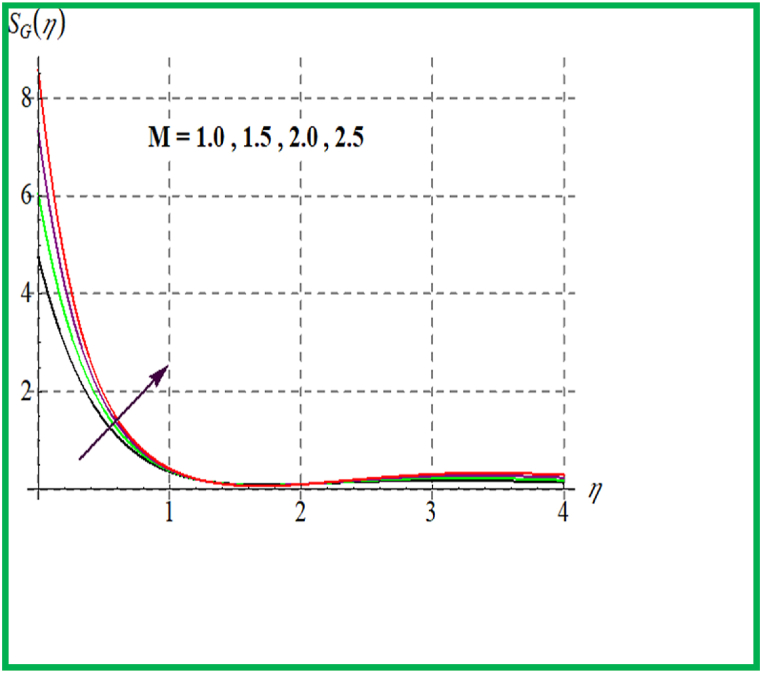
M versus $N_{G}$.

**Fig. 17:**
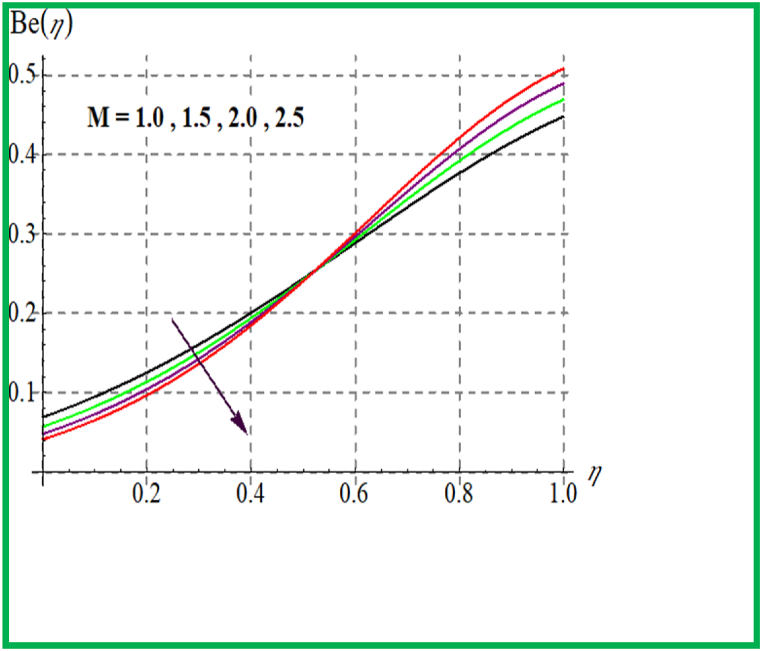
M versus Be.

**Fig. 18:**
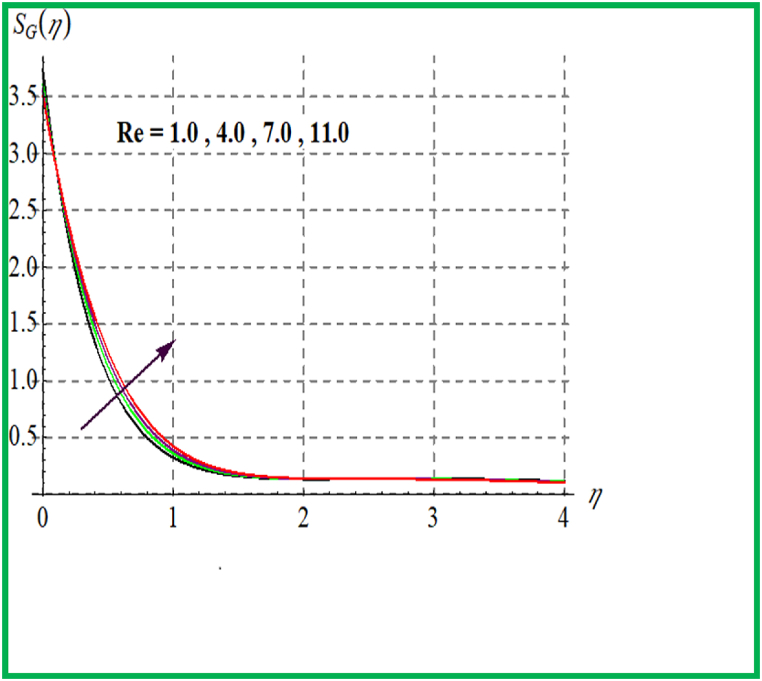
Re versus $N_{G}$.

**Fig. 19:**
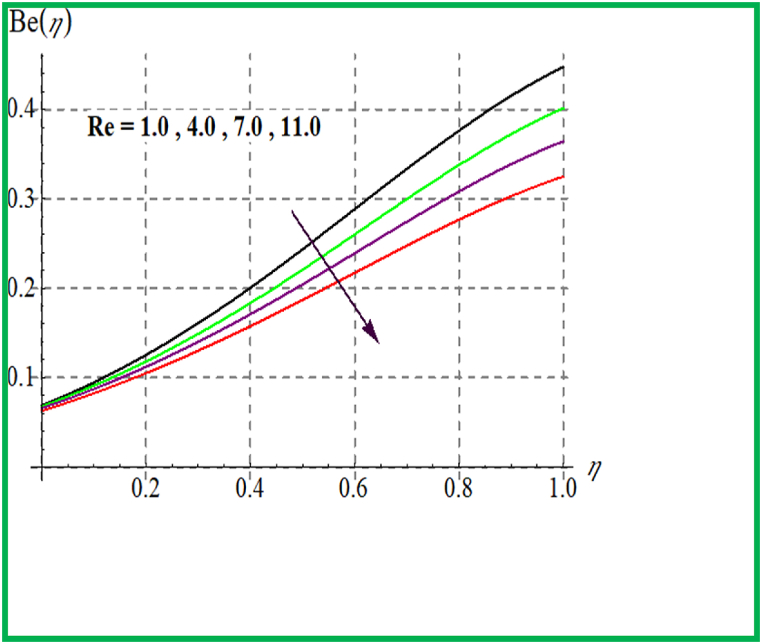
Re versus Be.

Fig. 14, Fig. 15 depict the influence of Br on $S_{G}$ and Be. For larger Br values $S_{G}$ upsurges. In fact, that larger Br more heat in the system is produced thus disorder in the system increases due to which $S_{G}$ increases. Larger Br corresponds to reduces the Bejan number (see Fig. 15). Fig. 16, Fig. 17 exhibit magnetic variable influence on Be and $S_{G}$. Physically inside liquid particles resistance increases for larger M which causes increment in disorder of system as result $S_{G}$ increases. For larger values of M Bejan number decreases as viscous effects are stronger then solutal and thermal transfer (see Fig. 17). Outcomes of Reynold number on Be and $S_{G}$ is revealed in Fig. 18, Fig. 19. It is observed that $S_{G}$ upsurges due to enlargement in Re values while opposite behavior is observed for Be.

### 7.5 Quantities of interest

#### 7.5.1 Wall shear force

Outcomes of $C_{fx}$ for various flow parameters is examined in Table 2. Larger magnetic and porosity parameters improve velocity gradient. An increment in $C_{fx}$ is noted for Reynold number.

**Table 2:** Computational values for $C_{fx}$.

Re	M	λ	$C_{fx}\text{.}$
1.0	1.0	0.2	1.49182
1.5			1.51017
2.0			1.52714
	1.0		1.49182
	1.5		1.7025
	2.0		1.86308
		0.2	1.49182
		0.4	1.56463
		0.6	1.63476

#### 7.5.2 Nusselt number

Computational results of $Nu_{x}$ for involved parameter are sketched in Table 3. Clearly noted that higher Prandtl number and radiation variable diminishes the temperature gradient. An enhancement in $Nu_{x}$ is seen for (Nb), (Nt) and (M).

**Table 3:** Numerical simulation for $Nu_{x}{\left({Re}_{x}\right)}^{-\frac{1}{2}}$.

R	Pr	Nb	Nt	M	$Nu_{x}$
1.0	1.0	0.5	0.5	1.0	0.174943
1.5					0.0691182
2.0					0.00587305
	1.5				1.49182
	2.0				1.49182
		0.6			0.180303
		0.7			0.185471
			0.6		0.180956
			0.7		0.186857
				1.5	0.234331
				2.0	0.276386

#### 7.5.3 Sherwood number

Variation of various sundry variable on mass transfer rate is examined in Table 4. Here it is noted that concentration gradient has opposite effect via (Nt) and (Nb). An improvement in $Sh_{x}$ is seen for Schmidt number

**Table 4:** Sherwood number $Sh_{x}{\left({Re}_{x}\right)}^{-\frac{1}{2}}$.

Nb	Nt	Sc	$Sh_{x}$
0.5	0.5	0.5	0.854252
0.7			0.744505
0.9			0.683138
	0.5		0.854252
	0.6		0.943833
	0.7		1.03575
		0.5	0.854252
		1.0	1.01725
		1.5	1.16495

## 8 Conclusions

The heat transfer improvement due Sutterby nanofluid subject to the entropy generation phenomenon. Additionally, the inspection has been predicted with viscous dissipation and radiative phenomenon. The shooting numerical technique us adopted for performing the simulations. The governing model under limiting case is verified. Some key observations are:

➢ The fluid viscosity declined for enhancing Deborah number and porosity parameter.
➢ The thermal profile gets reduction due to magnetic factor and Prandtl number.
➢ Higher random and thermophoresis motion variables lead to improve temperature.
➢ The concentration has reverse trend for random motion and thermophoresis variable.
➢ A decaying change in concentration profile is exhibited due to Schmidt number.
➢ The entropy generation phenomenon boosted due to larger Brinkman number and Reynolds number.
➢ The Bejan number decays due to increasing Reynolds number.

## Author contribution statement

Shuguang Li, Adel Bandar Alruqi: Conceived and designed the experiments.

M. Ijaz Khan: Performed the experiments; Wrote the paper.

Sami Ullah Khan: Performed the experiments; Analyzed and interpreted the data.

Sherzod Shukhratovich Abdullaev: Analyzed and interpreted the data

Bandar M. Fadhl, Basim M. Makhdoum: Contributed reagents, materials, analysis tools or data; Wrote the paper.

## Data availability statement

Data included in article/supplementary material/referenced in article.

## Declaration of competing interest

The authors declare that they have no known competing financial interests or personal relationships that could have appeared to influence the work reported in this paper.

## Nomenclature

p: pressure
$\left|\overset{•}{\gamma }\right|$: shear rate
m: power law index
L: gradient of velocity
x,y: Cartesian coordinates
$\mu_{f}$: dynamic viscosity
$\sigma_{f}$: electrical conductivity
$T_{w}$: wall temperature
$k_{1}$: mean absorption coefficient
τ: heat capacity ratio
$D_{T}$: thermophoresis coefficient
$C_{w}$: wall Concentration
$K_{r}^{2}$: reaction rate
$Q_{0}$: heat generation coefficient
De: Deborah number
M: magnetic variable
R: radiation parameter
$N_{b}$: Brownian motion variable
Ec: Eckert number
β: heat generation variable
$C_{fx}$: skin friction coefficient
$\tau_{w}$: shear stress
$q_{w}$: heat flux
$S_{G}$: entropy rate
L: diffusion variable
$\alpha_{2}$: concentration ratio variable
I: identity tensor
B: characteristics time
$A_{1}$: First Rivilin Erickson tensor
u,v: velocity components
$\rho_{f}$: density
$\nu_{f}$: kinematic viscosity
T: temperature
$\sigma^{*}$: Stefan Boltzmann constant
$T_{\infty }$: ambient temperature
$C_{p}$: specific heat
C: Concentration
$D_{B}$: Brownian motion coefficient
$C_{\infty }$: ambient concentration
$R_{1}$: molar gas constant
Re: Reynold number
Pr: Prandtl number
λ: porosity variable
Nt: thermophoresis parameter
γ: reaction variable
Sc: Schmidt number
$Nu_{x}$: Nusselt number
$Sh_{x}$: Sherwood number
$j_{w}$: mass flux
Br: Brinkman number
$\alpha_{1}$: temperature ratio variable
Be: Bejan number
